# miR-221-5p-Mediated Downregulation of JNK2 Aggravates Acute Lung Injury

**DOI:** 10.3389/fimmu.2021.700933

**Published:** 2021-11-25

**Authors:** Jing Yang, Hanh Chi Do-Umehara, Qiao Zhang, Huashan Wang, Changchun Hou, Huali Dong, Edith A. Perez, Marc A. Sala, Kishore R. Anekalla, James M. Walter, Shuwen Liu, Richard G. Wunderink, G.R. Scott Budinger, Jing Liu

**Affiliations:** ^1^ Department of Surgery, College of Medicine and University of Illinois Cancer Center, University of Illinois at Chicago, Chicago, IL, United States; ^2^ Division of Pulmonary and Critical Care Medicine, Department of Medicine, Feinberg School of Medicine, Northwestern University, Chicago, IL, United States; ^3^ Guangdong Provincial Key Laboratory of New Drug Screening, School of Pharmaceutical Sciences, Southern Medical University, Guangzhou, China; ^4^ State Key Laboratory of Organ Failure Research, Southern Medical University, Guangzhou, China; ^5^ Simpson Querrey Institute for Epigenetics, Feinberg School of Medicine, Northwestern University, Chicago, IL, United States

**Keywords:** JNK2, sepsis, lung inflammation and injury, micro RNA (miRNA), smARF, ubiquitination and degradation, mitochondrial dysfunction

## Abstract

Sepsis and acute lung injury (ALI) are linked to mitochondrial dysfunction; however, the underlying mechanism remains elusive. We previously reported that c-Jun N-terminal protein kinase 2 (JNK2) promotes stress-induced mitophagy by targeting small mitochondrial alternative reading frame (smARF) for ubiquitin-mediated proteasomal degradation, thereby preventing mitochondrial dysfunction and restraining inflammasome activation. Here we report that loss of JNK2 exacerbates lung inflammation and injury during sepsis and ALI in mice. JNK2 is downregulated in mice with endotoxic shock or ALI, concomitantly correlated inversely with disease severity. Small RNA sequencing revealed that miR-221-5p, which contains seed sequence matching to JNK2 mRNA 3’ untranslated region (3’UTR), is upregulated in response to lipopolysaccharide, with dynamically inverse correlation with JNK2 mRNA levels. miR-221-5p targets the 3’UTR of JNK2 mRNA leading to its downregulation. Accordingly, miR-221-5p exacerbates lung inflammation and injury during sepsis in mice by targeting JNK2. Importantly, in patients with pneumonia in medical intensive care unit, JNK2 mRNA levels in alveolar macrophages flow sorted from non-bronchoscopic broncholaveolar lavage (BAL) fluid were inversely correlated strongly and significantly with the percentage of neutrophils, neutrophil and white blood cell counts in BAL fluid. Our data suggest that miR-221-5p targets JNK2 and thereby aggravates lung inflammation and injury during sepsis.

## Introduction

Severe sepsis, a constellation of clinical signs of systemic inflammation combined with multiple organ dysfunction, is an important cause of death in the United States and the most common cause of death in medical ICUs (1–4). While improved primary source controls, which are primarily driven by early pathogen identification, appropriate antibiotic and organ supportive therapy, have reduced the incidence of multiple organ dysfunction and mortality from sepsis, the underlying pathobiology of sepsis remains poorly understood, and specific therapies to treat patients with sepsis are not available.

Acute lung injury (ALI) or acute respiratory distress syndrome (ARDS) secondary to sepsis is one of the leading causes of death in sepsis. ARDS, the most severe form of ALI, is a clinical syndrome defined by the acute onset of arterial hypoxemia refractory to low flow oxygen therapy and bilateral infiltrates on radiography (5–10). It is estimated that the incidence of ARDS is about 78.9/100,000 in the United States with a mortality rate of 40% (5–10). Even in those who survive ARDS, there is evidence that their long-term quality of life is unfavorably affected (5–10). Despite improvements in processes of care, including mechanical ventilation, fluid management, and other supportive care measures, the mortality from ARDS remains high (5–10), and specific and effective therapies are not available (5–10).

Sepsis and ARDS are linked to mitochondrial dysfunction, and mitochondrial defects have been extensively described in human subjects with sepsis- or severe pneumonia-associated ARDS as well as in animal models of ARDS (11–15). An established body of literature supports an association between mitochondrial damage and dysfunction and sepsis severity in murine models and in patients with sepsis (11, 12, 16–19). However, how sepsis and ARDS are linked to mitochondrial impair is not understood on the molecular level. Mitochondrial autophagy (mitophagy) is a selective form of autophagy that removes damaged mitochondria, thereby serving as an important mechanism of mitochondrial quality control (20, 21). Defective mitophagy results in accumulation of damaged mitochondria, which produce excessive mitochondrial reactive oxygen species (ROS) and release mitochondrial damage-associated molecular patterns (DAMPs) including damaged mitochondrial DNA (mtDNA) fragments into the cytoplasm (intracellular) and circulation (extracellular) that activate toll-like receptor 9 (TLR9) and the NLR family pyrin domain containing 3 (NLRP3) (previously known as NACHT, LRR and PYD domains-containing protein 3 [NALP3] and cryopyrin) inflammasome leading to excessive reactive species generation and exaggerated immune response (20–26). Accumulating evidence demonstrate dysregulated mitophagy in epithelial type II cells (AT2) and alveolar macrophages (AMs) in sepsis and severe acute lung injury (ALI)/ARDS in human subjects and animal models (27–30). Pharmacological and genetic manipulation of proteins involved in mitophagy has been reported to influence the outcomes in animal models of sepsis and ARDS (27–29, 31). However, the mechanisms underlying the deregulation of mitophagy and their functional significance in sepsis-induced ARDS remain largely unknown.

The c-Jun N-terminal protein kinase (JNK) is activated by environmental stresses to coordinate a host of fundamental cellular responses (32–35). JNK has two ubiquitously expressed isoforms, JNK1 and JNK2, which are highly homologous to each other (32–35). We and others have reported that JNK1 is the main JNK isoform activated by canonical JNK agonists while JNK2 activity is negligible and therefore most of the studies have been focused on JNK1 while the biological functions of JNK2 have been largely overlooked (35). We recently reported that JNK2 promotes stress-induced mitophagy independently of its kinase activity by targeting small mitochondrial ARF (smARF) for ubiquitin-mediated proteasomal degradation, thereby preventing mitochondrial dysfunction and restraining inflammasome activation (36). smARF is a short isoform of the tumor suppressor ARF that is translated from an internal initiation site Met45 and localizes exclusively to mitochondria (37–40). We reported that the loss of JNK2 led to accumulation of smARF, which in turn induced mitochondrial depolarization and excessive mitophagic and autophagic activity, resulting in lysosomal degradation of the mitophagy adaptor proteins, including p62 in the steady state. The depletion of p62 and other key components in the mitophagy machinery prevented the cells from mounting appropriate mitophagy in response to subsequent stress, leading to inflammasome hyperactivation and increased mortality during endotoxin shock (36). Here we report that the microRNA (miRNA) miR-221-5p targets JNK2 and thereby aggravates lung inflammation and injury during sepsis. Together with our previous report that JNK2 prevents mitochondrial dysfunction, our study might provide a potential mechanism for the well-documented association between mitochondrial dysfunction and sepsis.

## Results

### Loss of JNK2, but Not JNK1, Aggravates Lung Inflammation and Injury in Mouse Model of LPS-Induced Acute Lung Injury

**Figure 1:**
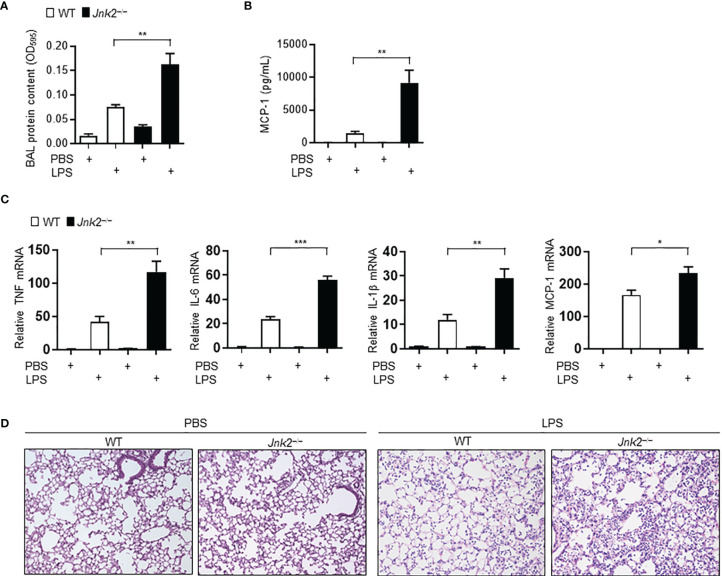
Loss of JNK2 aggravates lung inflammation and injury in mouse model of LPS-induced acute lung injury. Wild-type and JNK2 KO mice were subjected to i.t. LPS-induced ALI. Two days later, mice were harvested. **(A, B)** Protein content **(A)** and production of MCP-1 **(B)** in the BAL fluid. PBS: N=3-6. LPS: N=5-6. **(C)** mRNA expressions of inflammatory cytokines in whole lung homogenates as indicated. N=4. **(D)** Sectional lung histology from PBS- or LPS-treated wild-type and JNK2 KO mice. Data are presented as means ± sem. *p < 0.05; **p < 0.01; ***p < 0.001.

Sepsis is often associated with multiple organ dysfunction caused by dysregulation of host response to infection (1–4). The lung is among the most vulnerable and critical organs during sepsis, with acute lung injury (ALI) or ARDS being a common sepsis-induced inflammatory disorder (5–10). LPS, a component of Gram-negative bacterial endotoxin, is the main cause of ALI. We previously reported that loss of JNK2 rendered mice more susceptible to endotoxin-induced septic shock (36). We sought to determine the effect of JNK1 or JNK2 deficiency on lung inflammation and injury in mouse model of LPS-induced ALI. Wild-type, JNK1 KO or JNK2 KO mice were treated intratracheally with LPS, and we observed that the protein content was higher in the bronchoalveolar lavage (BAL) fluid of LPS-treated JNK2 KO mice compared to that in LPS-treated wild-type mice (**Figure 1A**), while it was not different between LPS-treated wild-type and JNK1 KO mice (**Supplementary Figure 1A**). The production of the potent inflammatory cytokine, MCP-1 (monocyte chemoattractant protein 1), was drastically augmented in the BAL fluid of LPS-treated JNK2 KO mice compared to LPS-treated wild-type mice (**Figure 1B**), while it was not different between LPS-treated wild-type and JNK1 KO (**Supplementary Figure 1B**). The mRNA expressions of inflammatory cytokines in the lung, including tumor necrosis factor (TNF), interleukin 6 (IL-6), IL-1β, and monocyte chemoattractant protein-1 (MCP-1), were also enhanced in LPS-treated JNK2 KO mice compared to LPS-treated wild-type mice (**Figure 1C**). On the other hand, there were no statistically significant differences in the production of TNF, interferon gamma (IFN-*γ*), IL-12p70, IL-6, or IL-10 in the BAL fluid of LPS-treated wild-type and JNK1 KO mice (**Supplementary Figure 1B**). Lung histology showed that the lungs from LPS-treated JNK2 KO had increased lung inflammation and injury, including increased inflammatory cell infiltration and thickening of the alveolar septa, as compared to LPS-treated wild-type mice (**Figure 1D**). Together, these data suggest that loss of JNK2, but not JNK1, exacerbates LPS-induced lung inflammation and injury in mice.

### Loss of JNK2 Worsens Lung Inflammation and Injury During *Pseudomonas* Pneumonia

**Figure 2:**
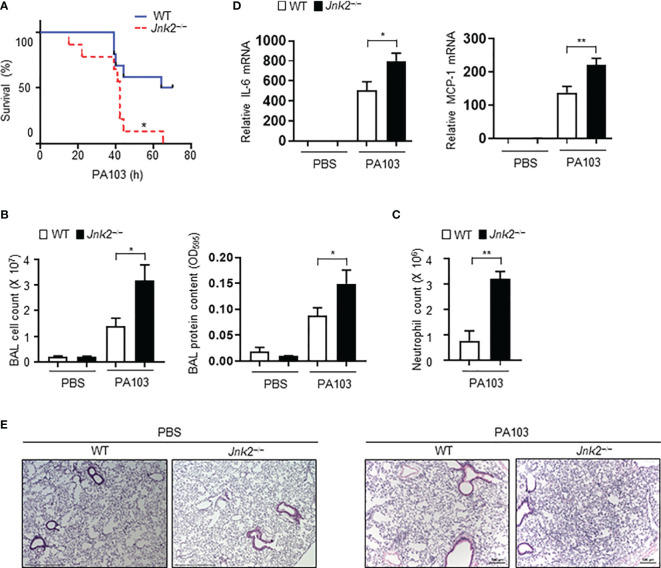
Loss of JNK2 worsens lung inflammation and injury during *pseudomonas* pneumonia. Wild-type and JNK2 KO mice were subjected to i.n. *pseudomonas aeruginosa* (strain PA103)-induced pneumonia. **(A)** Mortality of PA103-treated wild-type and JNK2 KO mice. N=9-10. **(B–E)** Cell count and protein content **(B)** and neutrophil count **(C)** in BAL fluid, and mRNA expressions of inflammatory cytokines from whole lung homogenates **(D)**, or sectional lung histology **(E)** from PBS- or PA103-treated mice. In **(B)**, PBS: N=3-8; PA103: N=13-14. In **(C)**, N=3-4. In **(D)**, PBS: N=3; PA103: N=4-5. Data are presented as means ± sem. *p < 0.05; **p < 0.01.

To further validate our conclusion that JNK2 deficiency exacerbates LPS-induced lung inflammation and injury, wild-type or JNK2 KO mice were intranasally infected with *Pseudomonas aeruginosa* (*P. aeruginosa*; strain PA103), a Gram-negative bacterium that produces the major virulence factor LPS. JNK2 KO mice had higher mortality compared to wild-type mice in response to PA103 infection (mice that did not die within 72 h recovered and survived; **Figure 2A**). The cell count and protein content were higher in the BAL fluid of PA103-treated JNK2 KO mice compared to that in PA103-treated wild-type mice (**Figure 2B**). Neutrophils are the major subset of infiltrating inflammatory cells in the lung in murine *Pseudomonas aeruginosa* pneumonia. We observed that neutrophil count in the BAL fluid was higher in PA103-treated JNK2 KO mice compared to PA103-treated wild-type mice (**Figure 2C**). mRNA levels of inflammatory cytokines, including IL-6 and MCP-1, were higher in PA103-treated JNK2 KO mice compared to PA103-treated wild-type mice (**Figure 2D**). Accordingly, histological examination revealed increased lung inflammation and injury, including increased inflammatory cell infiltration and thickening of the alveolar septa, in PA103-treated JNK2 KO mice compared to PA103-treated wild-type mice (**Figure 2E**). These data further support our conclusion that loss of JNK2 worsens lung inflammation and injury.

### JNK2 Deficiency Aggravates Lung Inflammation and Injury in Cecal Ligation and Puncture-Induced Sepsis

**Figure 3:**
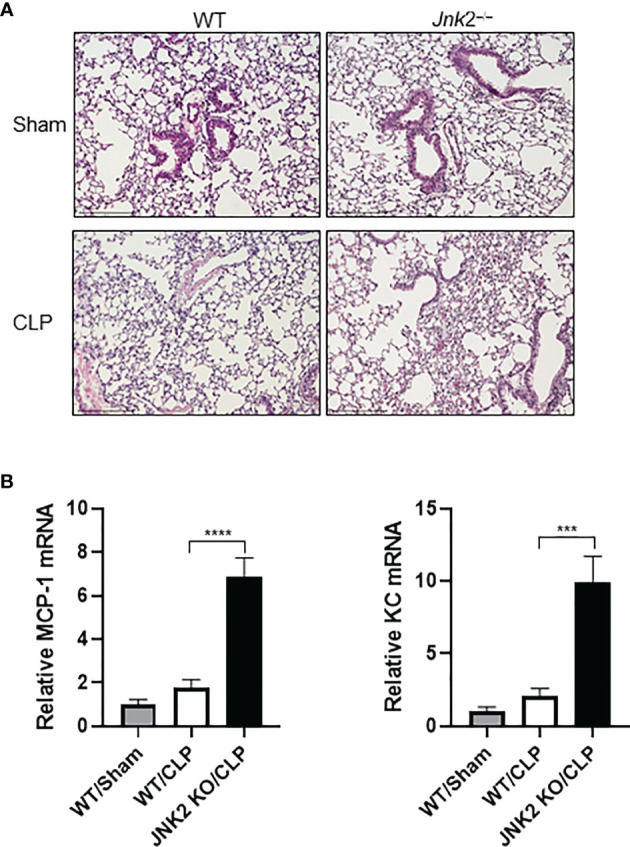
JNK2 deficiency augments lung inflammation and injury in CLP-induced sepsis. **(A)** Sectional lung histology of sham- or CLP-treated wild-type and JNK2 KO mice. **(B)** mRNA expressions of MCP-1 and KC from whole lung homogenates of sham-treated wild type mice or CLP-treated wild-type and JNK2 KO mice. N=3. Data are presented as means ± sem. ***p < 0.001; ****p < 0.0001.

Cecal ligation and puncture (CLP) is a widely used animal model of sepsis. We investigated whether JNK2 deficiency affects CLP-associated lung inflammation and injury. Indeed, lung histology showed increased lung inflammation and injury in CLP-treated JNK2 KO mice compared to CLP-treated wild-type mice, including increased inflammatory cell infiltration and thickening of the alveolar septa (**Figure 3A**). CLP-treated JNK2 KO mice also had increased expression of inflammatory cytokines, including MCP-1 and keratinocytes-derived chemokine (KC) in the lung compared to CLP-treated wild-type mice (**Figure 3B**). These data suggest that JNK2 deletion worsens CLP-associated lung inflammation and injury.

### JNK2 mRNA Levels Are Negatively Correlated With Disease Severity During Sepsis

**Figure 4:**
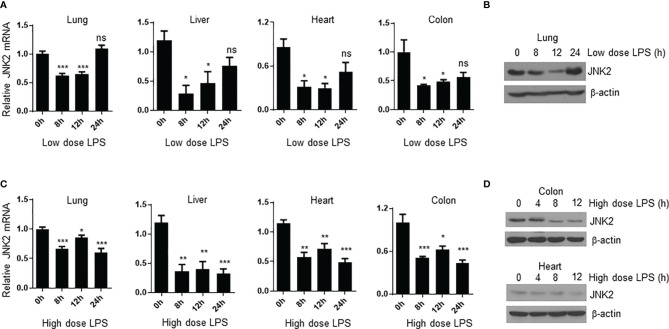
JNK2 mRNA levels are negatively correlated with disease severity during sepsis. **(A)** JNK2 mRNA expression in different organs of wild-type mice treated with low dose LPS for different times as indicated. **(B)** JNK2 protein expression in whole lung homogenates of wild-type mice treated with low dose LPS for different times as indicated. **(C)** JNK2 mRNA expression in different organs of wild-type mice treated with high dose LPS for different times as indicated. **(D)** JNK2 protein expression in whole colon or heart homogenates of wild-type mice treated with high dose LPS for different times as indicated. In **(A, C)**, N=3-6. Data are presented as means ± sem. *p < 0.05; **p < 0.01; ***p < 0.001; ns, not significant.

We were wondering whether JNK2 expression is subjected to regulation during endotoxin-induced septic shock. We observed that in mild endotoxic shock induced by intraperitoneal (i.p.) LPS (lower dose 12 mg/kg, which is non-lethal dose), JNK2 mRNA and protein levels in the lung were first decreased at 8-12 h after LPS treatment, and then increased and returned to baseline levels after 24 h (**Figures 4A, B**). Similar results were obtained in the other organs including liver, heart, and colon (**Figure 4A**). However, in severe septic shock (higher dose LPS 35mg/kg, which is lethal dose), JNK2 remained at lower levels and did not increase back at 24 h after the treatment (**Figures 4C, D**). We did not observe statistically significant decrease in JNK1 expression in the lung, colon, or liver under the same conditions (**Supplementary Figures 2A–C**). In contrast, JNK1 mRNA levels in the colon were modesty increased after LPS treatment (**Supplementary Figure 2B**). These data indicate the specific downregulation of JNK2 during septic shock.

### JNK2 mRNA Levels Are Negatively Correlated With Lung Injury Severity During *Pseudomonas* Pneumonia

**Figure 5:**
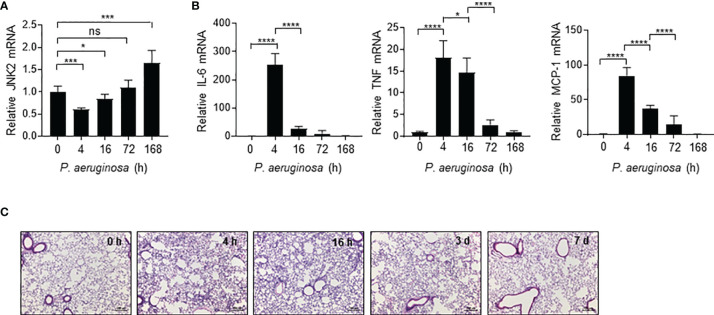
JNK2 mRNA levels are negatively correlated with lung injury severity during *pseudomonas* pneumonia. mRNA expressions of JNK2 **(A)** or inflammatory cytokines **(B)** in whole lung homogenates or lung histology by H&E staining **(C)** from wild-type mice treated with i.n. *pseudomonas aeruginosa* (strain PA103) for different times as indicated. N=3-6. Data are presented as means ± sem. *p < 0.05; ***p < 0.001; ****p < 0.0001; ns, not significant.

We observed that in response to intranasal *P. aeruginosa*, JNK2 mRNA levels in the wild-type mouse lungs were decreased at 4 h (**Figure 5A**), correlated with a robust induction of pro-inflammatory cytokines, including IL-6, TNF, and MCP-1 (**Figure 5B**), as well as lung inflammation and injury (**Figure 5C**). JNK2 mRNA levels were then increased at 16 h, returned to basal levels at 72 h, and were higher than baseline levels at day 7 (**Figure 5A**), correlated with a gradual decrease in pro-inflammatory cytokine expression (**Figure 5B**) and recovery from lung injury (**Figure 5C**). These data suggest that JNK2 mRNA levels are negatively correlated with lung injury severity during *pseudomonas* pneumonia.

### JNK2 mRNA Levels Are Also Negatively Correlated With Lung Injury Severity in LPS-Induced ALI Model

**Figure 6:**
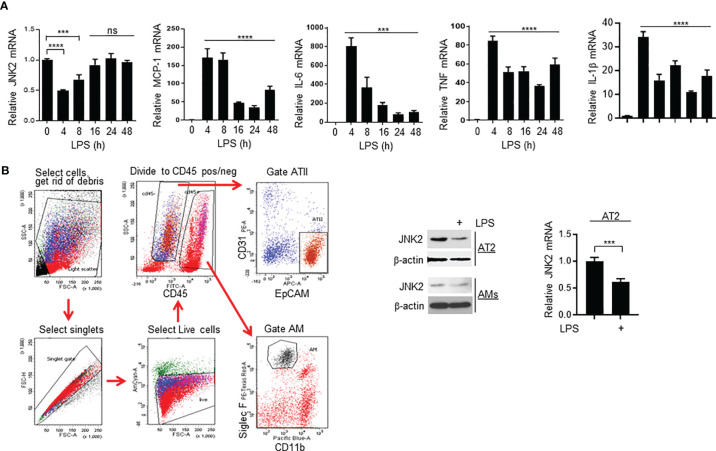
JNK2 mRNA levels are also negatively correlated with lung injury severity in LPS-induced ALI model. **(A)** mRNA expressions of JNK2 or inflammatory cytokines in whole lung homogenates of wild-type mice treated with i.t. LPS. **(B)** Left panel, gating strategy to flow sort AT2 cells and AMs from PBS-treated mouse lung. Middle panel, JNK2 protein expression in AT2 cells and AMs flow-sorted from PBS- or LPS-treated whole lung homogenates. Right panel, JNK2 mRNA level in AT2 cells flow-sorted from PBS- or LPS-treated mouse lung. N=3-6. Data are presented as means ± sem. ***p < 0.001; ****p < 0.0001; ns, not significant.

Similar to *P. aeruginosa*-induced ALI model, JNK2 mRNA levels in the wild-type mouse lungs were also decreased at 4 h after intratracheal LPS treatment (**Figure 6A**), correlated with a robust induction of pro-inflammatory cytokines, including MCP-1, IL-6, TNF, and IL-1β (**Figure 6A**). JNK2 mRNA levels were then increased at 8 h, and returned to basal levels after 16 h (**Figure 6A**), correlated with a gradual decrease in pro-inflammatory cytokine expression (**Figure 6A**). To identify the lung cell types in which JNK2 is downregulated by LPS, we isolated primary alveolar type 2 (AT2) cells and alveolar macrophages (AMs) by flow sorting as we previously reported (41) from PBS- and intratracheal LPS-treated mice (**Figure 6B**; left panel). We observed that JNK2 is downregulated in both AT2 cells and AMs by LPS (**Figure 6B**; middle and right panels).

### Small RNA Sequencing Revealed That miR-221-5p Is Upregulated in Response to LPS *In Vitro*, Resulting in JNK2 Downregulation

**Figure 7:**
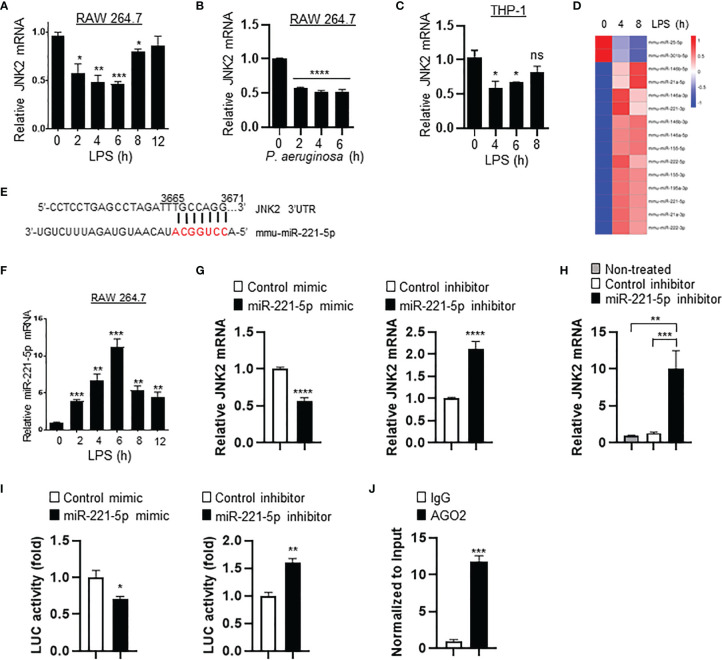
Small RNA sequencing revealed that miR-221-5p is upregulated in response to LPS *in vitro*, resulting in JNK2 downregulation. mRNA expressions of JNK2 in LPS- **(A)** or PA103-treated **(B)** RAW 264.7 cells, or LPS-treated THP-1 cells (PMA-differentiated) **(C)** as indicated. **(D)** Heatmap of differential expressed miRNAs in RAW 264.7 cells treated with LPS for 0, 4, and 8h. **(E)** Seed sequence of miR-221-5p matching to JNK2 mRNA 3’UTR. **(F)** Levels of miR-221-5p in RAW 264.7 cells treated with LPS for different times. **(G)** JNK2 mRNA levels in mouse lung epithelial cells (MLE-12) treated with mimic control or miR-221-5p mimic (left panel), or inhibitor control or miR-221-5p inhibitor (right panel). **(H)** JNK2 mRNA levels in whole lung homogenates of mice intratracheally treated without or with miRNA inhibitor control or miR-221-5p inhibitor. **(I)** JNK2 3’UTR luciferase activity in the presence of miR-221-5p mimic (left panel; normalized to control mimic) or miR-221-5p inhibitor (right panel; normalized to control inhibitor). **(J)** JNK2 mRNA in the RNA-protein complex using Ago2 or control IgG for IP in the presence of miR-221-5p mimic (normalized to input). Data are presented as means ± sem. *p < 0.05; **p < 0.01; ***p < 0.001; ****p < 0.0001; ns, not significant.

To investigate the mechanism by which JNK2 is downregulated by LPS, murine macrophage RAW 264.7 cells were treated with LPS (10 ng/ml) or *P*. aeruginosa (PA103, 20 multiplicity of infection). We observed that the mRNA levels of JNK2, but not JNK1, were decreased at 2-6 h after LPS treatment, then started to increase at 8 h and returned to baseline levels at 12 h in RAW 264.7 cells (**Figure 7A** and **Supplementary Figure 3A**). JNK2 mRNA levels were also reduced at 2-6 h after PA103 treatment in RAW 264.7 cells (**Figure 7B**). In human macrophages differentiated from human monocytic cell line THP-1 stimulated with phorbol-12-myristate-13-acetate (42), the mRNA levels of JNK2, but not JNK1, were also decreased at 4-6 h after LPS treatment, and then increased at 8 h, which were negatively associated with the induction of IL-6 dynamically (**Figure 7C** and **Supplementary Figures 3B, C**). To determine whether LPS-induced downregulation of JNK2 was due to reduced mRNA stability, RAW 264.7 cells were treated with LPS in the presence of RNA synthesis inhibitor actinomycin D (2 µM). LPS resulted in accelerated mRNA degradation rate of JNK2, but not JNK1, in the presence of actinomycin D (**Supplementary Figure 3D**). MicroRNAs (miRNAs) are small noncoding RNAs that interact with 3′-untranslated region (3’UTR) of target mRNAs leading to mRNA degradation and/or translational repression. Small RNA sequencing of LPS-treated RAW 264.7 cells revealed differentially expressed miRNAs by LPS (**Figure 7D** and **Supplementary Tables 1, 2**). Among them, miR-221-5p contains seed sequence matching to JNK2 but not JNK1 mRNA 3’UTR (**Figure 7E**), and importantly, it is induced by LPS with dynamically inverse correlation with JNK2 mRNA levels in LPS-treated RAW 264.7 cells (comparing **Figure 7F** and **Figure 7A**). Furthermore, miR-221-5p mimics, which are chemically modified double-stranded RNA molecules designed to mimic endogenous miRNAs, resulted in downregulation of JNK2 mRNA (**Figure 7G**, left panel). In contrast, miR-221-5p inhibitors, which are chemically-enhanced RNA oligonucleotides designed to bind and to sequester the complimentary, mature microRNA strand, resulted in upregulation of JNK2 mRNA (**Figure 7G**, right panel). To investigate the *in vivo* relevance of miR-221-5p-mediated downregulation of JNK2, miR-221-5p inhibitor or inhibitor control was delivered intratracheally into the mouse lung. As expected, JNK2 mRNA levels were markedly increased by miR-221-5p inhibitor as compared to inhibitor negative control (**Figure 7H**). These data suggest that miR-221-5p downregulates JNK2. To further evaluate whether miR-221-5p is functional in targeting JNK2 3’UTR, we performed JNK2 3′UTR Luciferase Reporter Assay. Transfection of miR-221-5p mimics inhibited while transfection of miR-221-5p inhibitors enhanced the luciferase reporter gene expression, which is controlled by JNK2 3′UTR in the promoter region (**Figure 7I**). miRNA-mediated silencing requires the interaction of the target mRNA with the miRNA and Argonaute (Ago) proteins, forming a miRNA-associated ribonucleoprotein complexes (RNPs), which is the core of the RNA-induced silencing complex (RISC) (43). Ago2 RNA immunoprecipitation (RIP) revealed the association of JNK2 mRNA with Ago2 in the presence of miR-221-5p mimics (**Figure 7J**). These data collectively suggest that miR-221-5p targets JNK2 mRNA.

### miR-221-5p Exacerbates Sepsis-Induced Lung Inflammation and Injury by Targeting JNK2 mRNA

**Figure 8:**
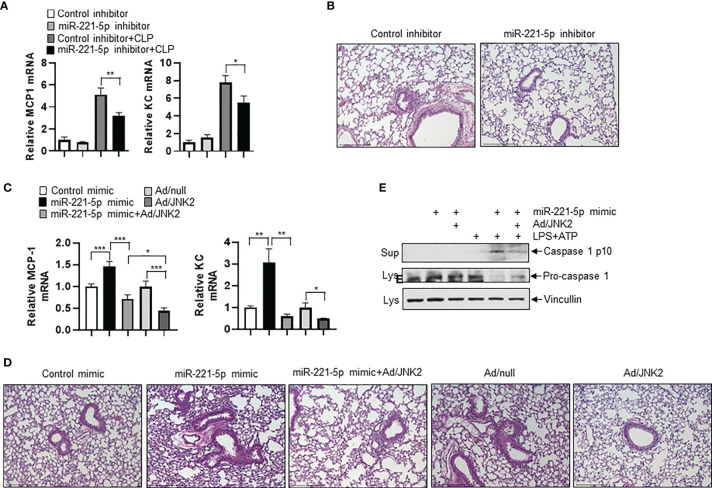
miR-221-5p exacerbates sepsis-induced lung inflammation and injury by targeting JNK2 mRNA. **(A, B)** mRNA levels of MCP-1 and KC in lung tissues **(A)**, or lung histology by H&E staining **(B)** from mice treated intratracheally with control inhibitor or miR-221-5p inhibitor, followed by sham or CLP treatment. N=4. **(C, D)** mRNA levels of MCP-1 and KC in lung tissues **(C)**, or lung histology by H&E staining **(D)** from mice treated intratracheally with control mimic, or miR-221-5p mimic, or miR-221-5p mimic with Ad/JNK2, or Ad/null, or Ad/JNK2, followed by CLP treatment. N=3-5. **(E)** Western blot of Caspase 1 p10 in the supernatant and Pro-caspase 1 in the cell lysates of RAW 264.7 cells transfected with control mimic or miR-221-5p mimic in the absence or presence of Ad/JNK2 infection, followed by treatment with LPS (10 ng/ml) for 16 h and then ATP (2mM) for 1 h. Data are presented as means ± sem. *p < 0.05; **p < 0.01; ***p < 0.001.

To determine the role of miR-221-5p in sepsis-induced lung inflammation and injury, we intratracheally delivered miR-221-5p inhibitor or inhibitor control, or miR-221-5p mimic or mimic control into the mouse lung, followed by CLP treatment. miR-221-5p inhibitor decreased the expression of CLP-induced inflammatory cytokines MCP-1 and KC (**Figure 8A**), and attenuated lung inflammation and injury (**Figure 8B**). In contrast, miR-221-5p mimic augmented the expression of CLP-induced MCP-1 and KC, and exacerbated lung inflammation and injury (**Figures 8C, D** and **Supplementary Figure 4A**), which was rescued by overexpression of exogenous JNK2 (**Figures 8C, D** and **Supplementary Figure 4B**). Additionally, overexpression of exogenous JNK2 by adenoviral delivery in the mouse lung resulted in attenuation of CLP-induced lung inflammation and injury as compared to empty adenovirus (Ad/null)-treated mice (**Figures 8C, D**). We previously reported that JNK2 promotes stress-induced mitophagy thereby preventing mitochondrial dysfunction and restraining the NLRP3 inflammasome activation (36). Activation of the NLRP3 inflammation results in the cleavage of pro-caspase 1 into active caspase 1, which in turn triggers the activation and release of interleukin1 (IL1) family proteins. We observed that miR-221-5p mimic increased the production of active, cleaved caspase 1 in LPS-primed, ATP-stimulated RAW 264.7 cells, as demonstrated by elevated levels of cleaved caspase 1 p10 and decreased levels of pro-caspase 1 proteins (**Figure 8E**). Again, overexpression of exogenous JNK2 rescued the effect of miR-221-5p mimic (**Figure 8E** and **Supplementary Figure 4C**). These data together suggest that miR-221-5p exacerbates sepsis-induced lung inflammation and injury by targeting JNK2 mRNA.

### JNK2 Levels in Alveolar Macrophages From Patients With Pneumonia Are Inversely Correlated With The Percentage Of Neutrophils, Neutrophil Count, and White Blood Cell Count in the BAL Fluid

**Figure 9:**
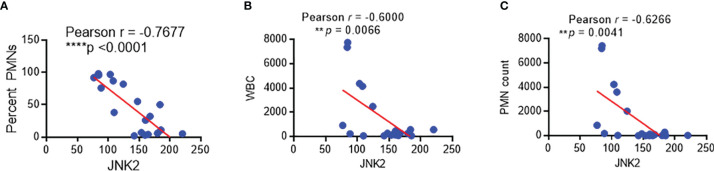
JNK2 levels in alveolar macrophages from patients with pneumonia are inversely correlated with the percentage of neutrophils, neutrophil count, and white blood cell count in the BAL fluid. Correlation of JNK2 mRNA levels (analyzed by RNA-seq) in AMs with neutrophil percentage **(A)**, WBC count **(B)** and neutrophil count **(C)** in the BAL fluid of patients with pneumonia. N=20 subjects. **p < 0.01; ****p < 0.0001.

We have developed protocols to reliably identify and isolate AMs by flow cytometry from non-bronchoscopic bronchoalveolar lavage (NBBAL) samples collected from mechanically ventilated patients with pneumonia (**Supplementary Figure 5A**) (44). This fluid was obtained as part of an Institutional Review Board-approved protocol to obtain an aliquot of lavage fluid from every NBBAL that is performed in the MICU. Flow-sorted AMs from 20 samples were obtained and high-quality RNA was extracted (**Supplementary Figure 5B**). RNA sequencing (RNA-Seq) was performed on these samples and high-quality transcriptomic data was obtained as measured by multiple variables (**Supplementary Figure 5C**). RNA-seq revealed that JNK2 mRNA levels in these AMs were inversely correlated strongly with the percentage of neutrophils, neutrophil count, and white blood cell (WBC) count in the BAL fluid of the patients with pneumonia (**Figures 9A–C**). Note, patient demographics and clinical characteristics were shown in **Supplementary Figure 5D**.

## Discussion

We and others have reported that JNK1 is the main JNK isoform activated by canonical JNK agonists while JNK2 activity is negligible and therefore most of the studies have been focused on JNK1 while the biological functions of JNK2 have been largely overlooked (35). Although there is well-established relationship between JNK and mitochondrial dysfunction and also the broad influence of JNK under conditions of stress and inflammation, those studies have been focused on JNK1 while the role of JNK2 is largely unknown (36). In contrast to JNK1, here we identified a protective role of JNK2 in ARDS. As JNK has many vital functions, specific targeting JNK2 without affecting the overall JNK activity (attributable to JNK1) highlights therapeutic potential. We also demonstrated that miR-221-5p targets JNK2 mRNA. As miRNAs are generally only about 22 nucleotides in length and can be released into different body fluids where they are remarkably stable, they offer potentials as biomarkers and drug therapies.

We show that JNK2 but not JNK1 mRNA levels are inversely correlated with lung injury severity in different mouse models of ALI induced by LPS, *Pseudomonas* pneumonia, or septic shock. Importantly, JNK2 mRNA levels in AMs from the BAL fluid of patients with pneumonia were inversely correlated strongly and significantly with the percentage of neutrophils, neutrophil and white blood cell counts in the BAL fluid. These data indicate that JNK2 might serve as a marker of sepsis severity and prognosis in the ICU.

An established body of literature supports an association between mitochondrial dysfunction and sepsis and ARDS severity in murine models and in patients with sepsis and ARDS. However, the underlying mechanism remains elusive. Several mechanisms have been proposed. For example, sepsis results in tissue hypoxia, which in turn affects oxidative phosphorylation. Abundant reactive species such as nitric oxide and superoxide are generated during sepsis, which affect mitochondrial respiration. Sepsis-induced hormonal changes also have impact on mitochondrial function. Genes encoding mitochondrial proteins have been reported to be downregulated during sepsis. Dysregulation of mitophagy and mitochondrial biogenesis are also proposed to explain the mitochondrial dysfunction in sepsis (29, 45–47). We previously reported that loss of JNK2 results in defective mitophagy, leading to mitochondrial dysfunction and robust inflammatory response. In the present study, we demonstrate that JNK2 is downregulated in ALI or septic shock, with dynamically inverse correlation with disease severity. Furthermore, JNK2 mRNA levels are also negatively correlated with neutrophil percentage in the BAL fluid of patients with pneumonia. Therefore, our study might provide a potential mechanism to explain the well-documented association between mitochondria dysfunction and sepsis/ARDS.

“Cytokine storm” refers to a prevalent hypothesis to explain the multiple organ dysfunction that develops with sepsis even after effective primary source control is achieved. According to this hypothesis, elevated levels of pro-inflammatory cytokines nonspecifically activate circulating and tissue-resident immune cells, which damage tissues and induce the release of more cytokines, creating a positive feedback loop that culminates in tissue damage and immune exhaustion (48, 49). While substantial evidence for this hypothesis can be found in patients with sepsis and animal models of sepsis, targeting this pathway with anti-cytokine therapy has been largely unsuccessful (48, 50). The newly emergent concept of “disease/tissue tolerance” refers to a complementary hypothesis to explain multiple organ dysfunction in sepsis (51–55). Tolerance is a host defense strategy that reduces the negative impact of infection on host fitness. Tolerance decreases the host susceptibility to tissue damage, or other fitness costs, caused by the pathogens or by the immune response against them, without directly affecting pathogen burden. According to this hypothesis, cells in different tissues can activate cell autonomous processes to maintain their function and survive even in an inflammatory milieu without affecting the severity of inflammation or pathogen burden. According to this model, the development of multiple organ dysfunction in sepsis represents a failure of tissue tolerance. The molecular basis for tissue tolerance is poorly understood, however, autophagy pathways and mitochondrial homeostasis have been implicated. For example, in mouse models of sepsis induced by cecal ligation and puncture (CLP), *Klebsiella pneumoniae*, or systemic LPS administration, a gain of tissue tolerance through activation of autophagy was found to be critical for host protection from sepsis (16, 55). The specific cellular basis for autophagy-mediated protection is not clear, however, a role in the removal of damaged mitochondria is suggested (17), and deregulated mitophagy has been recently recognized as a feature of severe sepsis and has been suggested to result in both enhanced inflammatory cytokine release and reduced tissue tolerance (54, 55). However, the signaling mechanisms that deregulate mitophagy to impair tissue tolerance during sepsis are unknown. Future studies are needed to investigate whether JNK2 servers as the molecular cue here.

## Materials and Methods

### Mice

*Jnk1* ^−/−^ and *Jnk2* ^−/−^ mice on the C57BL/6 background were kindly provided by Michael Karin (UCSD). The animal care and experiments were performed in compliance with the institutional and US National Institutes of Health guidelines and were approved by the Northwestern University Animal Care and Use Committee and the University of Illinois at Chicago Animal Care Committee. For the mortality studies, when mice became moribund (hunched posture, lack of curiosity, little or no response to stimuli and not moving when touched), a clinically irreversible condition that leads to inevitable death, according to the guidelines for the selection of humane endpoints in rodent studies at Northwestern University and University of Illinois at Chicago, they were sacrificed.

### Reagents

LPS (L2630), β-actin antibody (AC-15), and actinomycin D were from Sigma-Aldrich. ATP was from ENZO Life Sciences. JNK2 antibody was from Cell Signaling Technology (Cat # 4672). AGO2 antibody was from abcam (ab186733). Mouse miRIDIAN miR-221-5p mimic, miRIDIAN miRNA mimic negative control, miRIDIAN mouse miR-221-5p hairpin inhibitor, and miRIDIAN miNA hairpin inhibitor negative control were from Horizon Discovery. Ad/JNK2 was generated by ViraQuest, Inc. by cloning pCDNA3 Flag Jnk2a2 (Addgene, Item ID 13755) into the pVQAd CMV K-NpA vector. Ad/null was from ViraQuest, Inc. miR-221-5p inhibitor and inhibitor control, and miR-221-5p mimic and mimic control were from Horizon Discovery.

### LPS-Induced Lung Inflammation and Injury Model

Mice (male, 6–8 weeks of age) were given intratracheal instillation of LPS (6 mg per kg body weight) as we previously reported (56). After 48 h, the BAL fluid was collected for cell counts and protein quantification. Lungs were fixed, embedded in paraffin, and analyzed by staining with hematoxylin and eosin. In some experiments, lung tissues were collected for qRT-PCR of genes at different times after LPS treatment as indicated.

### Mouse Model of Acute Pneumonia

Mice (male, 6–8 weeks of age) were inoculated intranasally with *P. aeruginosa* (strain PA103, 2 × 10^5^ colony-forming units per mouse) as described as we previously reported (56). For survival experiments, mice were monitored every 8 h for up to 7 d. At 16 h after infection, the BAL fluid was collected for cell counts and protein quantification. Lungs were fixed, embedded in paraffin, and analyzed by staining with hematoxylin and eosin. In some experiments, lung tissues were collected for qRT-PCR of genes at different times after the infection as indicated.

### CLP-Induced Sepsis Model

CLP was performed as previously described (57). Briefly, mice were anesthetized with isoflurane, and a 1-cm midline abdominal incision was made. The cecum was then exposed, ligated and punctured with a 21-gauge needle. A small amount of cecal content was extruded from the perforation sites. The cecum was returned to the peritoneal cavity and the peritoneum is closed. Sham-operated mice were treated with cecal exposure without ligation and puncture. Mice were harvested at 24 h.

### Small RNA Sequencing

RAW 264.7 cells treated with LPS for 0, 4, and 8 h were subjected to small RNA-seq, which was performed by Novogene. Briefly, RNA integrity was assessed using Agilent Bioanalyzer 2100 system (Agilent Technologies, CA, USA), and RNA concentration was measured using Qubit^®^ 2.0 Flurometer (Life Technologies, CA, USA). Sequencing libraries were generated using NEBNext^®^Multiplex Small RNA Library Prep Set for Illumina^®^ (NEB, USA.) following the manufacturer’s instructions and index codes were added to attribute sequences to each sample. DNA fragments corresponding to 140~160bp (the length of small noncoding RNA plus the 3’ and 5’ adaptors) were recovered and the library quality was assessed using the Agilent Bioanalyzer 2100 system with DNA High Sensitivity Chips. The clustering of the index-coded samples was performed on a cBot Cluster Generation System using TruSeq SR Cluster Kit v3-cBot-HS (Illumia) according to the manufacturer’s instructions. After cluster generation, the library preparations were sequenced on an Illumina Hiseq 2500/2000platform and 50bp single-end reads were generated. The small RNA tags were mapped to reference sequence by Bowtie without mismatch to analyze their expression and distribution on the reference. miRNA expression levels were measured by TPM (transcript per million) using the normalization formula: Normalized expression = mapped read count/Total reads*1000000. Differential expression analysis of two conditions was performed using the DEGseq R package. P-value was adjusted using qvalue. qvalue<0.01 and |log2(foldchange)|>1 was set as the threshold for significantly differential expression by default.

### Lung Histology by Hematoxylin and Eosin Stain

Hematoxylin and Eosin (H&E) Staining was performed by the Research Histology Core at University of Illinois at Chicago (UIC). Briefly, 5-micron paraffin sections were deparaffinized and rehydrated. The sections were then stained in Mayers Hematoxylin for 1 minute, followed by wash with 4-5 changes of Tap water, 1X PBS for 1 minute, and 3 changes of distilled water. Slides were counterstained in Alcoholic-Eosin for 1 minute, and then dehydrated through 3 changes of 95% EtOH and 2 changes of 100% EtOH 1 minute each, followed by 3 changes of Xylene 1 minute each change and then mount and coverslip. Nuclei were stained blue while cytoplasm was stained pink.

### Analysis of Cytokines and Chemokines

The concentration of cytokines and chemokines in the BAL fluid were quantified by a cytometric bead array kit for mouse proinflammatory cytokines and chemokines (CBA; BD Biosciences).

### Adenovirus Infection

Mice were infected intratracheally with 1 × 10^9^ plaque-forming units of adenovirus per mouse. Cells were infected with adenovirus at a multiplicity of infection of 50.

### Intratracheal Delivery of miRNA Mimic or Inhibitor

miRNA mimic or inhibitor (1.25 µL of 0.4 nmol/µL) were mixed with 1.25 µL of invivofectamine-complexation buffer (cat# IVF3001, ThermoFisher), followed by addition of 2.5 µL of invivofectamine (pre-equilibrated to room temperature). The mixture was then vortexed vigorously for 2 s, and incubated at 50°C for 30 min, followed by addition of 45 µL of 1x PBS pH 7.4. The miRNA mimic or inhibitor solution was maintained at room temperature during the procedure. At 2 h, mice were harvested or underwent sham or CLP treatment.

### JNK2 3′UTR Luciferase Reporter Assay

JNK2 3’UTR reporter construct (LightSwitch™ 3’UTR GoClone^®^, Product code 32012, Active Motif Inc.), which expresses the luciferase reporter gene fused to the 3′UTR of JNK2, was transfected into HEK293 cells (ATCC) together with miR-221 mimic or mimic control (40 nM), or miR-221 inhibitor or inhibitor control (40 nM). At 48 h, luciferase activity was measured using LightSwitch™ Luciferase Assay Kit (Active Motif Inc.) according to the manufacturer’s instructions.

### Ago2 RNA Immunoprecipitation

Ago2 RIP was performed using Ago2 antibody for immunoprecipitation (IP) as described in: https://www.abcam.com/epigenetics/rna-immunoprecipitation-rip-protocol. JNK2 mRNA was detected by reverse transcriptase followed by qPCR using the following primers: 5’- AGTGATTGATCCAGACAAGCG-3’ and 5’-GCGGGGTCATACCAAACAGTA-3’, and was normalized to input.

### Quantitative PCR

Quantitative PCR (qPCR) was performed using iQ™ SYBR^®^ Green Supermix (BIO-RAD) on a CFX Connect™ Real-Time PCR Dection System (BIO-RAD). mRNA expression of a particular gene was normalized to hypoxanthine-guanine phosphoribosyltransferse (HPRT) for mouse genes. Primer sequences were listed in **Supplementary Table 3**. For qRT-PCR, total RNA was extracted using TRIzol^®^ (Thermo Fisher Scientific), followed by cDNA synthesis using M-MuLV Reverse Transcriptase according to the manufacturer’s instructions.

### miRNA Quantification by RT-qPCR

MiR-221-5p was quantified using stem-loop quantitative reverse transcription PCR (RT-qPCR). Briefly, RNA was extracted with miRNeasy Micro Kit (QIAGEN; Cat # 217084), followed by reverse transcription using stem-loop RT primers (Primer sequences were listed in **Supplementary Table 4**). The RT product was amplified by qPCR using miR-221-5p specific forward primer and the universal reverse primer (Primer sequences were listed in **Supplementary Table 4**).

### RNA-Seq of Alveolar Macrophages Isolated from BAL Fluid of Patients With Pneumonia

RNA-seq was performed in flow-sorted AMs from BAL fluid of patients as described as we reported (41).

### Fluorescence-Activated Cell Sorting

The lungs were digested with dispase (Corning) and 0.1 mg/mL DNase I. Red blood cells were removed using 1x BD Pharm Lyse solution (BD Biosciences). Cells were washed in MACS buffer (Miltenyi Biotech) and counted using a Countess automated cell counter (Invitrogen). For isolation of AT2 cells and AMs, single cell suspension was incubated in 0.5μg Fc Block (BD Biosciences) for 10 minutes at 4°C, followed by staining with FITC conjugated anti-mouse CD45 (eBioscience), APC conjugated anti-mouse EpCAM (eBioscience), PE conjugated anti-mouse CD31 (eBioscience), PE-CF594 conjugated anti-mouse SiglecF (BD Biosciences) and efluor450 conjugated anti-mouse CD11b (eBioscience). The FACS experiments were performed using a BD FACSAria SORP 5-Laser instrument (BD Immunocytometry Systems) equipped with 355nm, 405nm, 488nm, 561nm and 640nm excitation lasers located at the Northwestern University Flow Cytometry Core Facility. All data collection and sorting were performed using BD FACS Diva software (BD Biosciences) and data analyses were performed using FlowJo software (Tree Star, Ashland, OR). Fluorescence minus one (FMO) controls were used for gating analyses to distinguish positively from negatively staining cell populations. Compensation was performed using single color controls prepared from BD Comp Beads (BD Biosciences). Compensation matrices were calculated and applied using FlowJo software (Tree Star). Biexponential transformation was adjusted manually when necessary. For simultaneous isolation of AT2 and other myeloid cells, after blocking, cells were incubated with Biotin conjugated anti-mouse CD45 antibody for 10 minutes at room temperature. Cells then were separated using MagniSort Streptavidin Positive selection beads (eBioscience) according to the manufacturer’s instructions. AT2 cells will be isolated from the CD45 negative fraction by flow sorting using FITC conjugated anti-mouse CD45 (eBioscience), APC conjugated anti-mouse EpCAM (eBioscience) and PE conjugated anti-mouse CD31 (eBioscience). The myeloid cell populations will be isolated from the CD45 positive fraction by flow sorting using FITC conjugated anti-mouse CD45 (eBioscience), PerCPCy5.5 conjugated anti-mouse MHC II (BioLegend), eFluor450 conjugated anti-mouse Ly6C (eBioscience), APC conjugated anti-mouse CD24 (eBioscience), Alexa700 conjugated anti-mouse Ly6G (BD Biosciences), APCCy7 conjugated anti-mouse CD11b (BioLegend), PE conjugated anti-mouse CD64 (BioLegend), PE-CF594 conjugated anti-mouse SiglecF (BD Biosciences) and PECy7 conjugated anti-mouse CD11c (eBioscience) (58).

### Patients

Inclusion criteria: Mechanically ventilated adult patients aged 18 years or older in the ICU in whom a NBBAL was performed to investigate suspected pneumonia. Exclusion: NBBALs with > 12% bronchial epithelial cell, patients who were neutropenic, and who had bronchiectasis or cystic fibrosis. A non-bronchoscopic BAL (NBBAL) is a routine procedure performed in our medical intensive care unit (MICU) to sample the distal airspace in mechanically ventilated patients with suspected infection. During this bedside procedure, respiratory therapists (RT) advance a 16-French catheter *via* the patient’s endotracheal tube into the distal airways, where non-bacteriostatic saline is instilled and then withdrawn. The major difference between a traditional bronchoscopic BAL and a NBBAL is that an NBBAL is not performed under direct visualization. Rather, the NBBAL scope is introduced into either the left or right lung and advanced until resistance is encountered. NBBAL is routinely used in our MICU as it is inexpensive and can be performed by RTs without direct physician oversight facilitating timely alveolar sampling for patients admitted to the intensive care unit or who develop new acute lung pathology at night. The study protocol was approved by the Northwestern University Institutional Review Board (44).

### Statistical Analysis

Data were analyzed by an unpaired Student’s *t*-test, with the assumption of normal distribution of data and equal sample variance.

## Data Availability Statement

The raw data supporting the conclusions of this article will be made available by the authors, without undue reservation.

## Ethics Statement

The animal study was reviewed and approved by Northwestern University Animal Care and Use Committee and the University of Illinois at Chicago Animal Care Committee.

## Author Contributions

JY, HD-U, QZ, HW, CH, HD, EP, and MS performed experiments. KA and JW performed RNA-seq analysis of AMs flow-sorted from BAL fluid of patients. SL, RW, and GB provided reagents and suggestions. JL contributed to manuscript preparation, hypothesis generation, and experimental design. All authors contributed to the article and approved the submitted version.

## Funding

JL is supported by the US National Institutes of Health (HL141459 to JL).

## Conflict of Interest

The authors declare that the research was conducted in the absence of any commercial or financial relationships that could be construed as a potential conflict of interest.

## Publisher’s Note

All claims expressed in this article are solely those of the authors and do not necessarily represent those of their affiliated organizations, or those of the publisher, the editors and the reviewers. Any product that may be evaluated in this article, or claim that may be made by its manufacturer, is not guaranteed or endorsed by the publisher.
