# Supplementary material for: miR-221-5p-Mediated Downregulation of JNK2 Aggravates Acute Lung Injury
Source: Front Immunol. 2021 Nov 25;12:700933. doi: 10.3389/fimmu.2021.700933 (PMC8656235; doi:10.3389/fimmu.2021.700933)
Supplement: Supplementary file 1 [file DataSheet_1.pdf]

Supplementary Figure

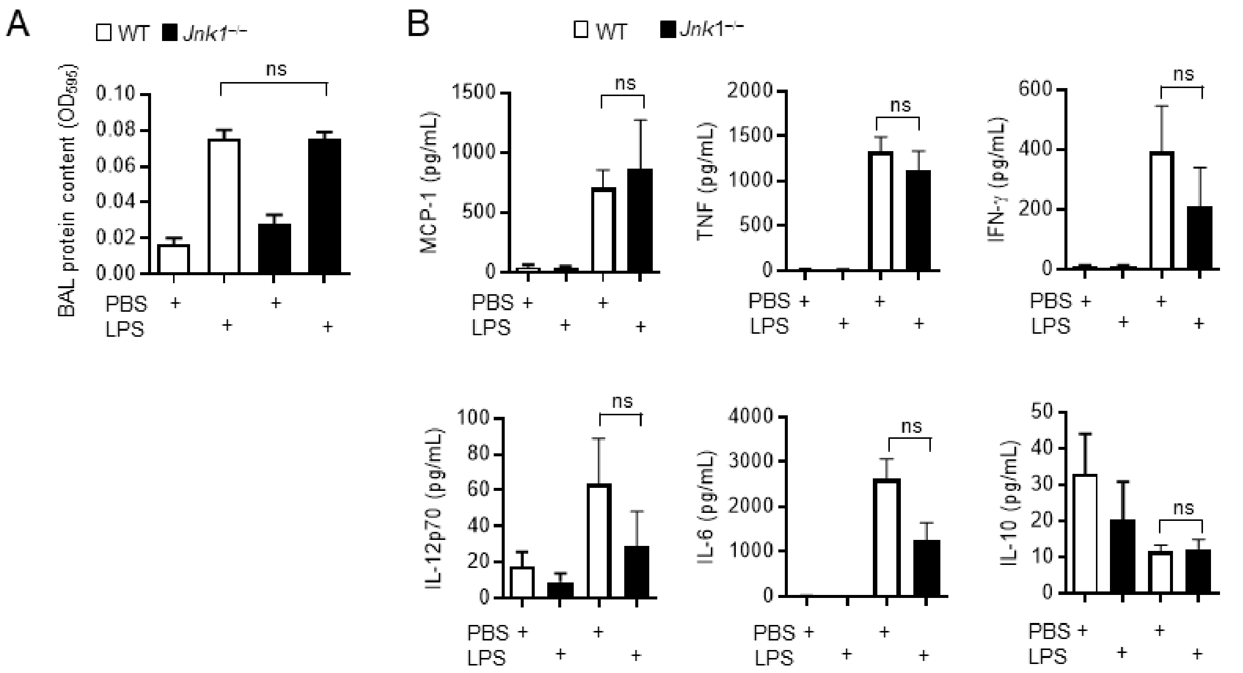

Supplementary Figure 1. Loss of JNK1 does not affect LPS-induced lung injury. (**A,B**) Protein content (**A**) and production of cytokines (**B**) in BAL fluid of PBS- or LPS-treated wild-type or JNK1 KO mice. Note, wild-type mice were the same as in **Fig. 1**. N=3-5. Data are presented as means±sem. ns, not significant.

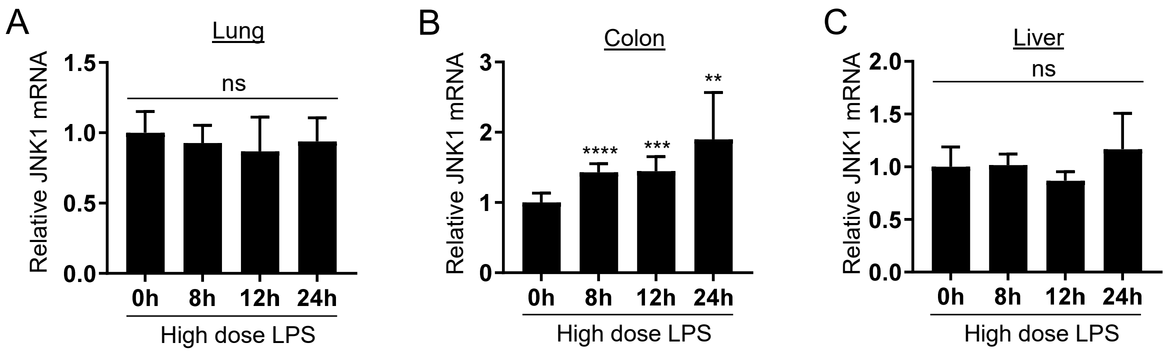

Supplementary Figure 2. LPS does not cause JNK1 downregulation in mice. JNK1 mRNA levels in whole tissue homogenates of lung, colon, or liver from wild-type mice treated with high dose LPS. N=4-5. Data are presented as means±sem. **, $p < 0.01$; ***, $p < 0.001$; ****, $p < 0.0001$; ns, not significant.

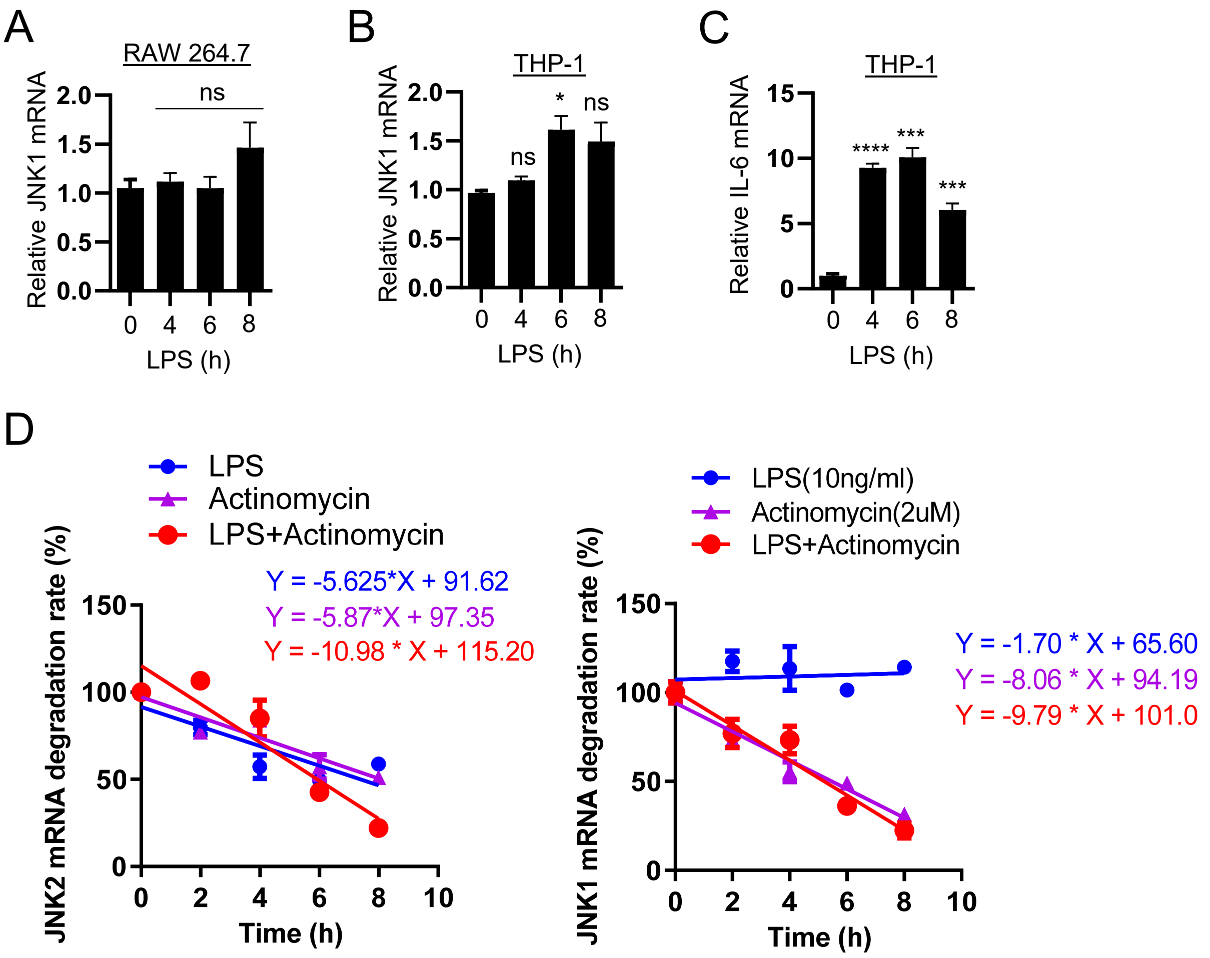

Supplementary Figure 3. LPS does not cause JNK1 downregulation in cells *in vitro* and LPS accelerates JNK2 mRNA degradation. JNK1 mRNA levels in LPS-treated RAW 264.7 cells (**A**) or PMA-differentiated THP-1 cells (**B**), or IL-6 mRNA levels in LPS-treated THP-1 cells (PMA-differentiated). Data are presented as means±sem. *, $p < 0.05$; ***, $p < 0.001$; ****, $p < 0.0001$; ns, not significant. (**D**) JNK2 and JNK1 mRNA degradation rate in LPS-, Actinomycin-, or LPS+Actinomycin-treated RAW 264.7 cells.

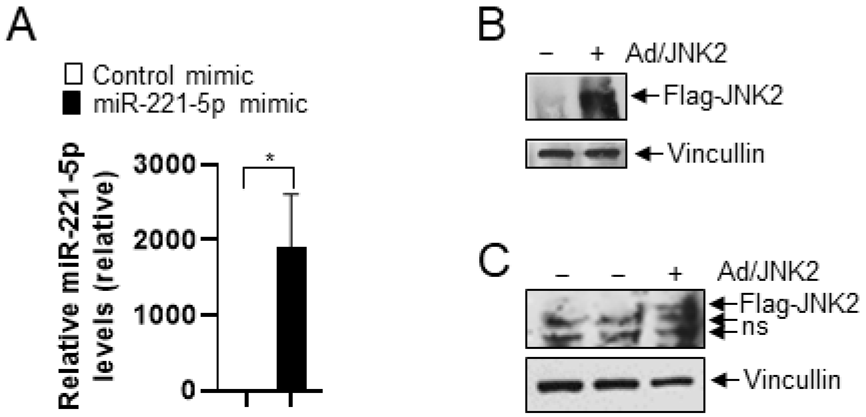

Supplementary Figure 4. miR-221-5p is overexpressed in the lung of mice intratracheally instilled with miR-221-5p mimic, and exogenous JNK2 is overexpressed in the lung of mice infected with Ad/JNK2 or RAW 264.7 cells infected with Ad/JNK2. (**A**) miR-221-5p levels in the lung of mice intratracheally instilled with control mimic or miR-221-5p mimic. N=5. *, $p < 0.05$. (**B**) Protein expression of exogenous Flag tagged-JNK2 in the lung of mice infected intratracheally with Ad/JNK2. (**C**) Protein expression of exogenous Flag tagged-JNK2 in RAW 264.7 cells infected with Ad/JNK2.

A

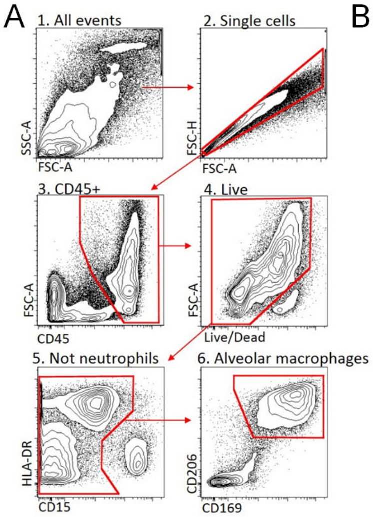

B

Sample #	# of Sorted Alveolar Macrophages	RNA Extracted (ng)	RNA Integrity #
1	15,000	9.76	9.1
2	17,000	13.72	9.2
3	22,000	21.50	8.7
4	47,000	33.40	6.3
5	2,800	2.80	8.7
6	30,000	25.00	9.3
7	1,200	2.48	10
8	20,000	14.90	9.6
9	1,000	2.10	9.2
10	14000	10.40	9.0
11	15,000	18.80	9.0
12	10,000	7.20	9.2
13	5,600	5.04	8.6
14	15,000	19.72	9.7
15	4,000	4.89	9.2
16	15,000	17.20	9.0
17	3,600	3.95	10
18	8,580	17.16	10
19	11,600	43.81	9.6
20	15,000	17.33	10

C

Quality of RNA-Seq Data Generated from FACS-Sorted Alveolar Macrophages

Quality Measure	Results
% of samples with a Quality score ≥30	96%
Read Depth	12.4M +/- 3.3M
Alignment	93.7% +/- 1.7%

D

Characteristic	Microbiologically Confirmed Pneumonia (n=20)
Age, mean (SD)	53 (14)
Male (%)	58
Suspected pneumonia type (%)	
▪ Community-acquired	10
▪ Health care-associated	20
▪ Hospital-associated	35
▪ Ventilator-associated	35
Antibiotics prior to NBBAL (%)	89
Days of mechanical ventilation prior to NBBAL, mean (SD)	5 (7)
Vasopressor use at the time of NBBAL (%)	44
In-Hospital Mortality (%)	32

Supplementary Figure 5. Gating strategy of AMs, RNA/RNA-seq quality control, and clinical characteristics of patients with pneumonia. (**A**) AMs were identified by flow cytometry as $CD11b^{+}HLADR^{++}CD206^{++}CD169^{+}FSC^{high}SSC^{high}$ cells. (**B**) RNA integrity of flow-sorted AMs from BAL fluid of patients with pneumonia. (**C**) RNA-seq quality control (QC) measured by Quality Score, read depth, and alignment. (**D**) Clinical characteristics of study patients. SD, standard deviation.
