# Supplementary material for: miR-221-5p-Mediated Downregulation of JNK2 Aggravates Acute Lung Injury
Source: Front Immunol. 2021 Nov 25;12:700933. doi: 10.3389/fimmu.2021.700933 (PMC8656235; doi:10.3389/fimmu.2021.700933)
Supplement: Supplementary file 4 [file Table_3.docx]

**Supplementary Table 3**

Mouse HPRT:

5'-AGGCCAGACTTTGTTGGATTTGAA-3'

5'-CAACTTGCGCTCATCTTAGGCTTT-3'

Mouse JNK2:

5’ - AGG TGG CGG ACT CAA CTT TC – 3’

5’ - CGA GTT CAC GGT AGG CTC TC – 3’

Mouse JNK1:

5’ - GTT CCC CGA TGT GCT TTT CC – 3’

5’- GGT GCT GGA GAG CTT CAT CT – 3’

Mouse TNF:

5'-GAACTGGCAGAAGAGGCACT-3'

5'-AGGGTCTGGGCCATAGAACT-3'

Mouse KC:

5’ - ACT GCA CCC AAA CCG AAG TC – 3’

5’ - TGG GGA CAC CTT TTA GCA TCT T – 3’

Mouse IL-6:

5′-AGTTGCCTTCTTGGGACTGA-3′

5′-TCCACGATTTCCCAGAGAAC-3′

Mouse IL-1β:

5’ - GCC CAT CCT CTG TGA CTC AT – 3’

5’ - AGG CCA CAG GTA TTT TGT CG – 3’

Mouse MCP-1:

5’ - AGG TCC CTG TCA TGC TTC TG – 3’

5’ - TCT GGA CCC ATT CCT TCT TG – 3’

**Supplementary Table 4**

Stem-loop RT primers:

5’- GTCGTATCCAGTGCAGGGTCCGAGGTATTCGCACTGGATACGACACAGAAA -3’

MiR-221-5p specific forward primer:

5’ – GTACACCTGGCATACAATGTAGA – 3’

Universal reverse primer

5’-GTGCAGGGTCCGAGGT-3’
